# B cell costimulatory molecules as potential biomarkers in HAM/TSP

**DOI:** 10.1186/1742-4690-8-S1-A106

**Published:** 2011-06-06

**Authors:** Soraya M Menezes, Daniele Decanine, Ricardo Khouri, Anne-Mieke Vandamme, Bernardo Galvão-Castro, Johan Van Weyenbergh

**Affiliations:** 1 Rega Institute, K.U. Leuven, Leuven, 3000 Belgium; 2 LIMI, CPqGM-FIOCRUZ, Salvador-BA, 40210-760, Brazil; 3 LASP, CPqGM-FIOCRUZ, Salvador-BA, 40210-760, Brazil

## 

Current therapy in HAM/TSP includes antiretrovirals and immunomodulators, such as corticosteroids and interferons, with modest clinical benefit. Since there are no validated biomarkers to monitor disease severity (DS), progression or prognosis, we investigated the ex vivo expression of CD80 and CD86 as possible biomarkers, as well as the effect of IFN-α/beta on their in vitro expression in 16 Brazilian HAM/TSP patients and 15 healthy controls (HC). Flow cytometry quantification indicated significantly elevated expression of both CD80 and CD86 ex vivo in T (p=0.044 and p=0.0016) and B (p=0.0003 and p=0.0057) cells, as compared to HC. In B cells, the ex vivo percentage of CD80+ positively correlated to DS (p=0.0083, r=0.78), but not age or disease duration, while ex vivo expression of CD80 was gender-biased, being significantly higher in women (p=0.028). Upon in vitro culture, CD80+ (p=0.037) and CD86+ lymphocytes significantly expand (p<0.0001), as compared to ex vivo levels, in patients only. In vitro treatment of patients’ PBMCs with IFN-beta, but not IFN-α resulted in significant stimulation of B cell CD86 expression (p<0.05). In HC, both IFN-α and IFN-beta brought about a significant increase in CD86 expression (p<0.05 and p<0.001). Neither IFN-α nor IFN-beta was able to significantly induce B cell CD80 expression in HC or patients. Consequently, B cell CD86/CD80 ratio was significantly increased upon stimulation with IFN-beta in both HC and patients. In conclusion, we propose novel biomarkers for possible clinical use in HAM/TSP trials: ex vivo CD80+ B cells positively correlating to DS and CD86+ B cells preferentially induced by IFN-beta.

